# Estimates of Illicit Opioid Use in the US

**DOI:** 10.1001/jamahealthforum.2025.0809

**Published:** 2025-05-09

**Authors:** David Powell, Mireille Jacobson

**Affiliations:** 1 RAND, Arlington, Virginia; 2 University of Southern California, Los Angeles

## Abstract

**Importance:**

Illicit opioids, particularly illicitly manufactured fentanyl (IMF), are major contributors to overdose deaths in the US. Understanding the prevalence and characteristics of illicit opioid use is crucial for addressing the opioid crisis.

**Objective:**

To estimate the prevalence of illicit opioid use, including IMF, and initial opioid exposure among those reporting illicit opioid use.

**Design, Setting, and Participants:**

This cross-sectional study was conducted using an online survey with targeted demographic quotas from June 10, 2024, to June 17, 2024. A total of 1515 participants aged 18 years and older from the US completed the survey. The analysis was conducted between June 30, 2024, and February 13, 2025.

**Main Outcomes and Measures:**

The primary outcome was self-reported illicit opioid use within the past 12 months. Secondary outcomes included initial exposure to opioids and perceived likelihood of overdose. Logistic regression was used to analyze associations with demographic and geographic factors.

**Results:**

A total of 1515 respondents completed the survey, including 770 female individuals (50.8%), 20 American Indian or Alaska Native (1.3%), 101 Asian or Pacific Islander (6.7%), 215 Black (14.2%), 1087 White (81.7%), and 24 multiracial (1.6%); 186 (12.3%) were aged 18 to 24, 242 (16.0%) 25 to 34, 327 (21.6%) 35 to 44, 280 (18.5%) 45 to 54, 281 (18.5%) 55 to 64, 139 (9.2%) 65 to 74, and 60 (4.0%) 75 to 84 years. Among this sample, 166 (10.96%; 95% CI, 9.38%-12.52%) reported nonprescription opioid use within the past 12 months, including 114 (7.52%; 95% CI, 6.20%-8.85%) of the 1515 respondents reporting IMF use. Among those reporting nonprescription opioid use within the past 12 months, 65 (39.16%; 95% CI, 31.73%-46.58%) reported that their first opioid use involved opioids prescribed to them, whereas 60 (36.14%; 95% CI, 28.84%-43.45%) reported that their first use involved prescription opioids not prescribed to them. Only 41 (24.70%; 95% CI, 18.14%-31.26%) answered that their first exposure involved illicit opioids. Seventy-one (4.69%; 95% CI, 3.62%-5.75%) of all respondents reported that it was very likely they would have an overdose due to opioid use. This rate increased to 33.33% (95% CI, 24.68%-41.99%) among those who had used IMF within the past 12 months. Illicit opioid use was higher among men, Black respondents, and younger age groups.

**Conclusions and Relevance:**

The findings of this cross-sectional study indicate a higher prevalence of illicit opioid use than previously reported, highlighting the need for more timely and accurate data to inform policy and intervention strategies. Enhanced data collection efforts are essential for understanding and mitigating the opioid crisis.

## Key Points

**Question:**

What is the prevalence of illicit opioid use, including illicitly manufactured fentanyl (IMF) use, in the US?

**Findings:**

In this national survey of 1515 adults conducted in June 2024, 166 respondents (11.0%) reported illicit opioid use and 114 (7.5%) reported IMF use within the past 12 months. These rates are much higher than previously reported estimates.

**Meaning:**

This study underscores the importance of improved data collection methods to effectively address the opioid crisis because national estimates of rates of illicit opioid use are typically released with a considerable lag time and may be underreported.

## Teaser

This cross-sectional survey study estimates the prevalence of illicit opioid use, including illicitly manufactured fentanyl, and initial opioid exposure among those reporting illicit opioid use.

## Introduction

Initially driven by prescription opioids, the opioid crisis transitioned to heroin in the early 2010s and then to illicitly manufactured fentanyl (IMF) a few years later.^1,2^ As the opioid crisis continues to evolve and even as polysubstance deaths become increasingly common,^3,4,5^ IMF is still involved in most overdose deaths.^6,7^ Despite the importance of illicit opioids in the current substance use landscape, little is known about the prevalence of illicit opioid use.

Although outcomes related to illicit opioid use, such as overdose^7,8,9^ and infectious disease,^10,11,12,13^ are important, they do not fully capture patterns and rates of use. Understanding the current size of the illicit opioid market is critical for forecasting treatment need and assessing the value of regulatory and policy decisions such as making naloxone available over the counter or distributing fentanyl test strips.^14,15,16^ Furthermore, such information is needed to gauge the harms associated with illicit opioid use and the extent to which use is pervasive, risky, or both.

The literature has struggled to determine population rates of illicit opioid use, often relying on samples targeting specific populations.^17,18,19,20,21,22^ Data from nationally representative surveys, such as the National Survey on Drug Use and Health (NSDUH)^23,24^ or others,^25^ historically do not measure the prevalence of IMF use. The NSDUH only recently asked about IMF, reporting that 0.3% of the population aged 18 years or older used IMF in the past year for 2022.^26^ In addition, information on initial opioid use among people reporting illicit use is scant, except for specific populations.^27,28^ As the opioid crisis has evolved, attention to the role of prescription opioids has waned, even though overdose deaths from prescription opioids remain high and prescription opioids may operate as critical pathways to illicit opioid use.

Prior work has criticized the use of surveys to determine heroin use rates,^29^ suggesting that the true rate of chronic heroin use in the US is more than 16 times the rate reported by the NSDUH.^30,31^ Hypotheses for why NSDUH rates are likely too low include sampling frame omissions and underreporting, but little evidence is available to evaluate these premises or serve as a useful benchmark. Given the few available estimates of illicit opioid use, especially IMF use, and concerns about existing surveys, additional data points are needed.

Resources that permit researchers and policymakers to track illicit opioid trends in a timely manner and report on them with minimal lag time are critical. Quicker turnaround times heighten their relevance to the current substance use environment, especially given the pace of the evolution of the opioid crisis. The importance of more timely data has been emphasized by researchers and policymakers,^32,33^ but few resources to improve the timeliness of available information have been identified and tested.

This study endeavored to provide new and timely information on illicit opioid use among a national population. We developed and fielded a survey using an online platform designed for empirical research and used demographic quotas to match the national population. One advantage of this platform is that researchers can conduct surveys and report outcomes in a short time frame. Most research reports heroin and IMF use from years prior to publication. This survey study was conducted in June 2024 and asked respondents about opioid misuse and illicit opioid use in the past 12 months and self-assessed likelihood of experiencing an opioid overdose. Those self-reporting illicit opioid use were asked about initial use of opioids.

## Methods

### Data and Survey Design

We developed and fielded a survey to a panel from Respondi, an online platform often used in academic research due its high-quality nationally representative panels.^34,35,36,37,38,39,40,41,42,43^ Respondi actively recruits panelists, who are required to go through a double opt-in process, and continuously checks for active engagement with the surveys. Participants in the panel are invited to take a survey. They are not told the subject of the survey to avoid concerns about respondent selection. This study received approval from RAND’s Human Subjects Protection Committee and the University of Southern California’s institutional review board and followed the American Association for Public Opinion Research (AAPOR) reporting guidelines. We informed participants of the sensitive nature of the survey and required online consent before survey completion (eMethods, Section 1.1 in Supplement 1).

Respondents were asked to self-report demographic information from multiple choice questions. The demographic questions captured gender, age, education, race (Asian American or Pacific Islander, American Indian or Alaska Native, Black, White, or Other), ethnicity, and state of residence. The survey enforced recruitment quotas for those completing the survey based on sex (male/female), race (White/not White [American Indian/Alaska Native, Asian American or Pacific Islander, Black, and other races were all combined into “non-White race” due to expected small sample sizes]), age-group (ages 18-29, 30-39, 40-49, 50-59, 60 years and older), and region (Northeast, Midwest, South, West). Any respondents from outside of the US or younger than 18 years were screened out. Once a quota was reached, subsequent respondents were screened out if they reported a geographic or demographic characteristic that matched a fully met quota category. The survey remained in the field until 1515 surveys were completed, a number predetermined by the cost of each respondent and the project budget.

Participants were asked about use of nonprescription opioids within the past 12 months, with heroin and IMF given as examples and could respond in 1 of 3 ways: (1) yes, I intentionally used illicit opioids; (2) yes, I may have unintentionally used illicit opioids; or (3) no. Respondents choosing 1 or 2 were subsequently asked about IMF use within the past 12 months with the following 3 options: (1) yes, I intentionally used illicitly made fentanyl; (2) yes, I may have unintentionally used illicitly made fentanyl; or (3) no.

Respondents answering that they had used illicit opioids in the past 12 months were also asked whether their first use of opioids involved (1) opioids prescribed to you; (2) prescription opioids not prescribed to you; or (3) illicit opioids. All respondents were asked to rate their likelihood of experiencing an overdose as (1) unlikely; (2) somewhat likely; (3) likely; or (4) very likely.

The survey was piloted among 200 respondents and redesigned based on response patterns and feedback; pilot data were not included in the final sample. Respondents were given an attention check question in the middle of the survey. Participants who responded incorrectly exited the survey before completing it (746 of 2290 respondents who initiated the survey). In addition, 29 respondents who did not complete the survey were excluded from the analysis. The survey remained in the field until we received 1515 completed surveys, corresponding to a survey period of June 10, 2024, to June 17, 2024. We checked for internal consistency of the responses (eMethods, Section 1.2 in Supplement 1).

### Statistical Analysis

We calculated the percentage of participants selecting each response and estimated 95% CIs. We used logistic regression to analyze the characteristics associated with (1) illicit opioid use and (2) IMF use. The explanatory variables were male (female and self-identify excluded); Black and White (all others excluded); Hispanic; ages 18 to 34 years and ages 35 to 54 years (ages ≥55 years excluded); South, West, and Northeast (Midwest excluded). We reported probability changes associated with each characteristic, holding the values of all other characteristics constant. 95% CIs were estimated using robust standard errors and the delta method. Statistical significance was set at 2-tailed *P* < .05. Analyses were conducted using Stata statistical software (version 17.0; Stata Corp). The analysis was conducted between June 30, 2024, and February 13, 2025.

## Results

### Sample Characteristics

We received 1515 completed surveys. Table 1 provides the sample characteristics. The survey sample included 770 (50.8%) reporting female sex, 1087 (71.7%) identifying their race as White, and 327 (21.6%) reporting their age as between 35 and 44 years. Overall, 560 respondents (37.0%) reported living in a state in the South census region. We compared demographic and geographic characteristics with corresponding rates from the May 2024 Current Population Survey (CPS) (eTable in Supplement 1). The CPS is the primary source of monthly labor force statistics in the US and is nationally representative of the noninstitutionalized population. Our survey sample was similar to the CPS in the distribution by gender, race, ethnicity, and census region. The age distribution was also similar, although our sample had a smaller share of people ages 75 years or older. The largest differences between our sample and the CPS related to educational attainment because our sample was more likely to have attended college.

**Table 1.** Summary Statistics of Survey Sample

Characteristic	No. (%)
	National sample (N = 1515)
Sex	
Female	770 (50.8)
Male	739 (48.8)
Self-identify	6 (0.4)
Education (ages ≥25 y)	
High school or less	360 (23.8)
Some college	310 (20.5)
AA degree or trade school	238 (15.7)
BA degree	407 (26.9)
Graduate degree	200 (13.2)
Race	
American Indian/Alaska Native	20 (1.3)
Asian American and Pacific Islander	101 (6.7)
Black	215 (14.2)
White	1087 (71.7)
Other race	68 (4.5)
Multiracial	24 (1.6)
Ethnicity	
Hispanic	256 (16.9)
Age group, y	
18-24	186 (12.3)
25-34	242 (16.0)
35-44	327 (21.6)
45-54	280 (18.5)
55-64	281 (18.5)
65-74	139 (9.2)
75-84	60 (4.0)
Political party	
Republican	553 (36.5)
Democratic	628 (41.5)
Other	334 (22.0)
Region	
Northeast	276 (18.2)
Midwest	328 (21.7)
South	560 (37.0)
West	351 (23.2)

### Illicit Opioid Use Rates

Among those in our sample, 166 (10.96%; 95% CI, 9.38%-12.53%) reported nonprescription opioid use within the past 12 months, including 117 (7.72%; 95% CI, 6.38%-9.07%) reporting intentional nonprescription opioid use with the other 49 (3.23%; 95% CI, 2.34%-4.13%) reporting unintentional nonprescription opioid use (Table 2).

**Table 2.** Illicit Opioid Use Within the Past 12 Months Among 1515 Participants^a^

Variable	% (95% CI)	
	Illicit opioid use	Illicit fentanyl use
Intentional or unintentional use	10.96 (9.38-12.53)	7.52 (6.20-8.85)
Intentional use	7.72 (6.38-9.07)	4.95 (3.86-6.04)
Unintentional use	3.23 (2.34-4.13)	2.57 (1.78-3.37)

^a^ Table includes rates of reporting nonprescription opioid use (the first column) and illicitly manufactured fentanyl use (the last column) within the past 12 months overall and by whether respondents reported that this use was intentional or unintentional. The last 2 rows of estimates add up to the top row.

Most of those with nonprescription opioid use reported illicit fentanyl use. For the full sample, 114 (7.52%; 95% CI, 6.20%-8.85%) reported IMF use within the previous 12 months. Seventy-five (4.95%; 95% CI, 3.86%-6.04%) reported intentional IMF use, whereas 39 (2.57%; 95% CI, 1.78%-3.37%) reported unintentional IMF use.

### Initial Opioid Exposure Among Participants Reporting Illicit Opioid Use

Among respondents reporting nonprescription opioid use within the past 12 months, 65 (39.16%; 95% CI, 31.73%-46.58%) reported their first use of opioids involved opioids prescribed to them, 60 (36.14%; 95% CI, 28.84%-43.45%) reported their first use involved prescription opioids not prescribed to them, and 41 (24.70%; 95% CI, 18.14%-31.26%) answered that their first exposure to opioids involved illicit opioids (Table 3).

**Table 3.** Initial Opioid Exposure Among Respondents Reporting Illicit Opioid Use^a^

Variable	% (95% CI)	
	Illicit opioid use	Illicit fentanyl use
**Initial exposure**		
Opioids prescribed to you	39.16 (31.73-46.58)	40.35 (31.34-49.36)
Prescription opioids not prescribed to you	36.14 (28.84-43.45)	33.33 (24.68-41.99)
Illicit opioids	24.70 (18.14-31.26)	26.32 (18.23-34.40)
**Intentional use**		
Opioids prescribed to you	40.17 (31.29-49.05)	45.33 (34.07-56.60)
Prescription opioids not prescribed to you	38.46 (29.65-47.28)	33.33 (22.66-44.00)
Illicit opioids	21.37 (13.94-28.79)	21.33 (12.06-30.60)
**Unintentional use**		
Opioids prescribed to you	36.73 (23.24-50.23)	30.77 (16.28-45.25)
Prescription opioids not prescribed to you	30.61 (17.71-43.52)	33.33 (18.54-48.13)
Illicit opioids	32.65 (19.52-45.78)	35.90 (20.84-50.95)

^a^ Responses to “When you first used opioids for medical or for nonmedical purposes, were they…” are reported with 95% CIs. For the top set of results, n = 166 for sample reporting illicit opioid use within the past 12 months; n = 114 for sample reporting fentanyl use within the past 12 months. For the middle set of results, n = 117 for intentional illicit opioid use; n = 75 for intentional fentanyl use. For the bottom set of results, n = 49 for unintentional illicit opioid use; n = 39 for unintentional fentanyl use.

Among those reporting fentanyl use within the past 12 months, 46 (40.35%; 95% CI, 31.34%-49.36%) responded that their first opioid exposure was opioids prescribed to them, 38 (33.33%; 95% CI, 24.68%-41.99%) from prescription opioids not prescribed to them, and 30 (26.32%; 95% CI, 18.23%-34.40%) from illicit opioids.

We disaggregated first opioid use by whether in the past year illicit opioid use was intentional. Among respondents reporting intentional nonprescription opioid use, 47 (40.17%; 95% CI, 31.29%-49.05%) reported that their first use of opioids involved opioids prescribed to them; 45 (38.46%; 95% CI, 29.65%-47.28%) reported that their first use involved prescription opioids not prescribed to them; and 25 (21.37%; 95% CI, 13.94%-28.79%) reported that their initial exposure involved illicit opioids. Among respondents reporting intentional fentanyl use, 34 (45.33%; 95% CI, 34.07%-56.60%) reported that their first use of opioids involved opioids prescribed to them; 25 (33.33%; 95% CI, 22.66%-44.00%) reported that their first use involved prescription opioids not prescribed to them; and 16 (21.33%; 95% CI, 12.06%-30.60%) reported that their first use involved illicit opioids.

Rates of initial exposure differed for those with unintentional illicit opioid use, with a higher proportion reporting initial use of illicit opioids. Among respondents reporting unintentional nonprescription opioid use within the past 12 months, 18 (36.73%; 95% CI, 23.24%-50.23%) reported that their initial use of opioids involved opioids prescribed to them; 15 (30.61%; 95% CI, 17.71%-43.52%) reported that their first use involved prescription opioids not prescribed to them; and 16 (32.65%; 95% CI, 19.52%-45.78%) reported that their first use involved illicit opioids. For respondents reporting unintentional IMF use, 12 (30.77%; 95% CI, 16.28%-45.25%) reported that their first use involved opioids prescribed to them; 13 (33.33%; 95% CI, 18.54%-48.13%) reported that their first use involved prescription opioids not prescribed to them; and 14 (35.90%; 95% CI, 20.84%-50.95%) reported that their first exposure involved illicit opioids.

### Self-Reported Likelihoods of Overdosing

Among the full sample, 1277 (84.29%; 95% CI, 82.46%-86.12%) claimed it was unlikely that they might overdose from opioid use, whereas 71 (4.69%; 95% CI, 3.62%-5.75%) reported that it was very likely (Figure, A). In contrast, only 54 (32.53%; 95% CI, 25.40%-39.66%) of those with nonprescription opioid use within the past 12 months responded that it was unlikely that they might overdose from opioids, whereas 40 (24.10%; 95% CI, 17.59%-30.60%) said that it was very likely (Figure, B). Among those with fentanyl use within the prior 12 months, 20 (17.54%; 95% CI, 10.56%-24.53%) reported it was unlikely that they would overdose from opioids; 38 (33.33%; 95% CI, 24.68%-41.99%) responded that it was very likely (Figure, C).

**Figure.**
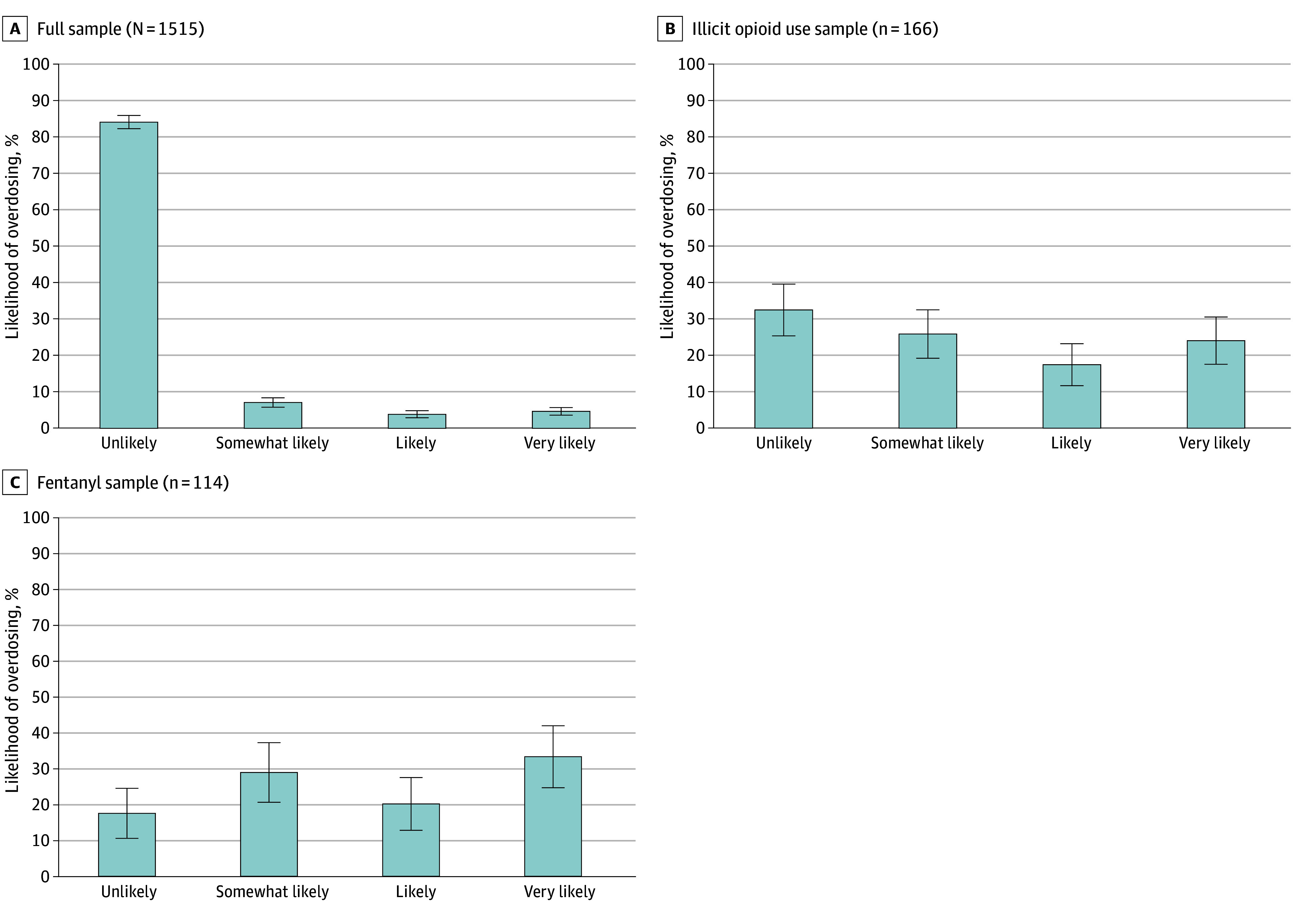
Self-Reported Likelihood of Overdosing From Opioid Use by Sample Answers to the question, “How likely do you think it is that you might overdose from opioid use?” Whiskers indicate the 95% CIs.

### Characteristics Associated With Illicit Opioid Use

Table 4 shows estimated probability differences for illicit opioid and IMF use associated with demographic and geographic variation. We estimated that illicit opioid use within the past 12 months was 5.4 (95% CI, 2.2-8.5) percentage points higher for men, whereas IMF use was 4.4 (95% CI, 1.6-7.2) percentage points higher. For Black respondents, the rate of illicit opioid use was 6.6 (95% CI, 1.9-11.2) percentage points higher and IMF use was 4.2 (95% CI, 0.4-8.1) percentage points higher than the baseline group (all other racial groups). White respondents had lower use rates, although the estimates were not statistically different from the baseline group.

**Table 4.** Multivariable Analyses of Factors Associated With Illicit Opioid Use Within the Past 12 Months and Illicitly Made Fentanyl Use Within the Past 12 Months^a^

Variable	Estimated probability difference relative to excluded category (95% CI)	
	Illicit opioid use within past 12 mo	Illicitly made fentanyl use within past 12 mo
Total No.	1515	1515
Male	0.054 (0.022 to 0.085)	0.044 (0.016 to 0.072)
Race and ethnicity		
Black	0.066 (0.019 to 0.112)	0.042 (0.004 to 0.081)
White	−0.032 (−0.073 to 0.009)	−0.032 (−0.066 to 0.002)
Hispanic	0.055 (0.019 to 0.091)	0.040 (0.008 to 0.071)
Age, y		
18-34	0.237 (0.155 to 0.320)	0.174 (0.095 to 0.254)
35-54	0.214 (0.132 to 0.297)	0.161 (0.083 to 0.240)
Region		
South	0.019 (−0.025 to 0.063)	0.011 (−0.026 to 0.048)
West	0.017 (−0.032 to 0.067)	−0.010 (−0.053 to 0.033)
Northeast	0.010 (−0.043 to 0.063)	−0.010 (−0.056 to 0.035)

^a^ Estimates represent the change in probability of the event associated with each characteristic, estimated jointly using a logistic regression model. 95% CIs were estimated using robust standard error estimates and the delta method. The excluded categories for male are female and self-identify. The excluded categories for Black and White are all other reported racial groups (including those reporting more than one race). The excluded category for age is the age 55 years or older age group. For region, the excluded category is the Midwest.

Hispanic respondents had 5.5 (95% CI, 1.9-9.1) percentage point higher rates of illicit opioid use and 4.0 (95% CI, 0.8-7.1) percentage point higher rates of IMF use. Compared with the excluded group (ages ≥55 years), respondents aged 18 to 34 years had higher rates of illicit opioid use by 23.7 (95% CI, 15.5-32.0) percentage points and higher rates of IMF use by 21.4 (95% CI, 13.2-29.7) percentage points. Illicit opioid use among respondents aged 35 to 54 years was higher by 17.4 (95% CI, 9.5-25.4) percentage points; IMF use was higher by 16.1 (95% CI, 8.3-24.0) percentage points. We did not find statistically significant geographic differences, possibly because the study was not powered to detect such differences.

## Discussion

The opioid crisis represents one of the greatest public health challenges of our time, yet estimates of illicit opioid use are rare and typically available only years after data collection, limiting our ability to monitor trends in prevalence. This survey study estimated near real-time rates of illicit opioid use.

These findings suggest that illicit opioid use and, in particular, IMF use is more prevalent than previously estimated. Almost 11% of the population aged 18 years and older reported using illicit opioids within the past 12 months, including 7.5% of the population aged 18 years and older using IMF.

Research critical of prior survey evidence on illicit substance use estimated that the true rate of chronic heroin use was more than 16 times the rate reported in the NSDUH.^30^ Our estimate of IMF use is 25 times as large as the 2022 NSDUH rate for those aged 18 years or older (7.5% vs 0.3%).^26^ Assuming that illicit opioid use has increased, these differences are consistent with prior estimates of NSDUH undercounts of illicit opioid use.^31^

It is unclear why the NSDUH rates are so much lower, but 2 key survey differences may contribute. First, NSDUH estimates are generated from 2 questions: “Have you ever, even once, used illegally made fentanyl?” Respondents answering “yes” are then asked, “How long has it been since you last used illegally made fentanyl?” Although variations in wording may lead to different estimates, it remains unclear why these changes would result in such markedly divergent results. Second, about half of NSDUH surveys are conducted in-person,^44^ which may also affect responses. Additional research is needed to test these hypotheses.

Many people reporting illicit fentanyl use (40%) initially used prescription opioids prescribed to them. A smaller but still large share initially used prescription opioids prescribed to someone else. However, 26% of people reporting illicit fentanyl use responded that their initial opioid exposure was with illicit opioids. These rates varied based on whether the nonprescription opioid use within the past 12 months was intentional or unintentional. The rate of first use of illicit opioids was only 21% among those reporting intentional fentanyl use, but rose to 36% among those reporting unintentional fentanyl use. These results suggest the continuing importance of prescription opioids in the current opioid crisis, despite the disproportionate share of overdose deaths involving illicit opioids. Policies targeting prescription opioid dispensing and general cultural shifts regarding opioid prescribing can still play a role in ameliorating or exacerbating the opioid crisis. Prescribing policies continue to merit meaningful attention from both policymakers and researchers.

In addition, the survey distinguished between intentional and unintentional illicit opioid use, a distinction that has not been made in nationally representative samples despite widespread concerns that fentanyl is often added to other drugs.^45,46,47^ Comparing the unintentional illicit opioid use rate to the total illicit opioid use rate, these survey results found that 29.5% of illicit opioid use was unintentional, whereas 34.2% of IMF use was unintentional. These percentages are high, implying high penetration of IMF into illicit drug markets more broadly. For comparison, a study^48^ of medically attended opioid overdoses in San Francisco, Calfornia, found that 42.7% of illicit opioid use was unintentional.

Self-reported assessments of the likelihood of overdosing vary substantially based on past illicit opioid use. Whereas 4.7% of the population reported that it was very likely that they will overdose from opioids, this rate increased to one-third among those with fentanyl use within the past 12 months. Overall, 17.4% of people reporting fentanyl use thought that it was unlikely that they would overdose from opioid use, implying that most people using IMF recognize the heightened risk of overdose from such consumption. Although speculative, the implied awareness about risk suggest that this population may be receptive to interventions that reduce the likelihood of overdose.

These results can be used to help assess risks associated with IMF use. According to the Centers for Disease Control and Prevention (CDC), an estimated 64 410 overdose deaths in the US involved synthetic opioids (excluding methadone) between July 2023 and June 2024—a period that aligns with the 12-month window for our survey’s fentanyl use question.^49^ This figure corresponds to a synthetic opioid overdose death rate of 19.0 per 100 000 US residents.^50^ Our 7.5% estimate of illicit fentanyl use is for adults aged 18 years and older. If we conservatively assume no fentanyl use among the 21.5% of the population that is younger than 18 years,^51^ the national illicit fentanyl use rate was 5.9% and implies an annual overdose death rate of 0.32% among the population using illicit fentanyl.

### Limitations

This cross-sectional study has important limitations. First, although the survey sample matched national demographic shares, it may not be representative on unobserved dimensions. In particular, Respondi panelists are required to have internet access, which may alter the sample in systematic ways. Consistent with this, we observed higher educational attainment in our sample relative to the CPS. This bias would likely lead us to underestimate illicit opioid use.^52^ Second, the survey asked about initial opioid exposure for those reporting illicit opioid use within the past 12 months, but we cannot claim that initial exposure caused subsequent illicit opioid use. Third, the survey did not ask about use of other substances, so we cannot capture polysubstance use. Fourth, all information was self-reported. Our attention check and analysis of the internal consistency of responses (eMethods, Section 1.2 in Supplement 1) provided confidence that respondents were actively engaged with the survey. However, reporting bias concerns remain. Fifth, by including the option “Yes, I may have unintentionally used illicit opioids,” individuals who had used an illicit substance but were unsure whether it contained fentanyl could have selected this response. This uncertainty may have artificially inflated our estimates of overall illicit opioid use. However, it would be otherwise difficult to ascertain unintended use. We presented intentional and unintentional rates separately partially due to this issue. Finally, we did not track trends or attribute differences in illicit opioid use to specific policies. The results should be understood as characterizing recent illicit opioid use while future data collection can provide evidence on trends and policy impacts.

## Conclusions

Despite the importance of and widespread interest in understanding the prevalence of illicit opioid use in the US, efforts to assess this information in a timely manner are limited. This study fielded a survey that could be part of a national toolkit to quickly and repeatedly monitor illicit opioid prevalence at low cost. The findings of this study suggest that illicit opioid use may be more common than previously reported. Although there are trade-offs in how information has been collected in prior surveys relative to this study, our results are consistent with reports indicating substantial underreporting of illicit opioid use in ongoing national surveys. The data presented herein should be treated as a substantive data point for understanding and curtailing the ongoing opioid crisis. More near-real-time data are needed to evaluate not only where we are in the epidemic but, more importantly, whether we are making progress in reining it in.

## Notes

Supplement 1. eMethods eFigure 1. Personal Impacts of Opioid Use Among Respondents with No Prescription or Illicit Opioid Use within Past 12 Months (N=1107) eFigure 2. Self-Reported Likelihood of Overdosing from Opioid Use for Respondents with No Prescription or Illicit Opioid Use within Past 12 Months and No Personal Impact from Opioid Use (N=1057) eTable. Demographic and Geographic Characteristics of the Survey Sample with Rates from the May 2024 Current Population Study

Supplement 2. Data Sharing Statement
