# Error in Results Section

**DOI:** 10.1001/jamahealthforum.2025.2563

**Published:** 2025-06-20

The Original Investigation titled “Estimates of Illicit Opioid Use in the US,”^1^ published online on May 9, 2025, was corrected to fix an error in the text of the Results section. Specifically, the sentence “The rate of intentional IMF use was 75 (4.95%; 95% CI, 3.86%-6.04%) reported intentional IMF use, whereas 39 (2.57%; 95% CI, 1.78%-3.37%) reported unintentional IMF use” was changed to “Seventy-five (4.95%; 95% CI, 3.86%-6.04%) reported intentional IMF use, whereas 39 (2.57%; 95% CI, 1.78%-3.37%) reported unintentional IMF use.” This article was corrected online.
