# Supplementary material for: Estimates of Illicit Opioid Use in the US
Source: JAMA Health Forum. 2025 May 9;6(5):e250809. doi: 10.1001/jamahealthforum.2025.0809 (PMC12065033; doi:10.1001/jamahealthforum.2025.0809)
Supplement: Supplement 1. — eMethods eFigure 1. Personal Impacts of Opioid Use Among Respondents with No Prescription or Illicit Opioid Use within Past 12 Months (N=1107) eFigure 2. Self-Reported Likelihood of Overdosing from Opioid Use for Respondents with No Prescription or Illicit Opioid Use within Past 12 Months and No Personal Impact from Opioid Use (N=1057) eTable. Demographic and Geographic Characteristics of the Survey Sample with Rates from the May 2024 Current Population Study [file jamahealthforum-e250809-s001.pdf]

Supplemental Online Content

Powell D, Jacobson M. Estimates of Illicit Opioid Use in the US. *JAMA Health Forum.* 2025;6(5):e250809. doi:10.1001/jamahealthforum.2025.0809

eMethods

eFigure 1. Personal Impacts of Opioid Use Among Respondents with No Prescription or Illicit Opioid Use within Past 12 Months (N=1107)

eFigure 2. Self-Reported Likelihood of Overdosing from Opioid Use for Respondents with No Prescription or Illicit Opioid Use within Past 12 Months and No Personal Impact from Opioid Use (N=1057)

eTable. Demographic and Geographic Characteristics of the Survey Sample with Rates from the May 2024 Current Population Study

This supplemental material has been provided by the authors to give readers additional information about their work.

© 2025 Powell D, et al. *JAMA Health Forum*.

eMethods.
Section 1.1: Introductory Text to the Survey

The first screen seen by participants included the following text:

This survey asks about your knowledge of and personal experiences with substance use in the United States. The survey is for a research study conducted by RAND and the University of Southern California. It includes sensitive questions about prior substance use. Your responses are confidential and the researchers receiving the data will not have access to any identifying information. Please do not include any personally identifying information in any of your responses. You can stop the survey at any time. We know that this information is personal, but your answers are important to our understanding of substance use in the US. This survey should take 10 minutes of your time.

If the topics in this survey bring up issues that you would like to discuss with someone. Please call the Substance Abuse and Mental Health Services (SAMHSA) helpline: 1-800-662-3457.

This study was approved by the RAND Human Subjects Protection Committee and has also been reviewed by the USC Institutional Review Board (IRB). The IRB is a research review board that reviews and monitors research studies to protect the rights and welfare of research participants. Contact the IRB if you have questions about your rights as a research participant or have complaints about the research. You can contact RAND's Human Subjects Protection Committee toll-free at (866) 697-5620 or by emailing hspcinfo@rand.org. When you contact the RAND Committee, please reference Study #2023-N0309. You can also contact USC's IRB at (323) 442-0114 or by email at hrpp@usc.edu. When you contact USC's IRB, please reference Study UP -24-00279.

Participants were then asked to check a box signifying that they wanted to proceed:

I've read the information above and agree to proceed with the survey

Section 1.2: Internal Consistency of Responses

We checked whether responses were internally consistent to safeguard against inattention and false responses. For our first test, we evaluated how people responded that they were personally impacted by opioids among respondents who self-reported no prescription opioid use in the past 12 months, no prescription opioid misuse in the past 12 months, and no use of non-prescription opioids in the past 12 months (N=1,107). Figure S1 reports this sample's responses regarding how they have been personally impacted by opioids. Respondents could select more than one option for this question. 95.48% (95% CI: 94.26% - 96.71%) reported no impact while a small share (3.25% [95% CI: 2.21% - 4.30%]) reported that they struggled with dependence and 1.72% (95% CI: 0.95% - 2.48%) reported that they sought treatment. These latter answers would be consistent with opioid dependence issues over 12 months before the survey. The low rates of respondents in this sample claiming any prior impacts of opioids to their life is consistent with the absence of opioid use and misuse within the past 12 months.

Next, we evaluated the self-reported rates of overdose likelihood among respondents who self-reported no prescription opioid use in the past 12 months, no prescription opioid misuse in the past 12 months, no use of non-prescription opioids in the past 12 months, and reported that they have not been personally impacted by opioids (N=1,057). These selections do not imply that the respondent has never had exposure to opioids, nor does it imply that they might not anticipate an overdose involving opioids if, for example, they frequently use illicit substances and are aware that illicitly-made fentanyl could potentially contaminate those substances. Figure S2 shows that 92.90% (95% CI: 91.36% - 94.45%) of these respondents reported it was unlikely that they would overdose from opioid use. This high rate suggests that the responses were internally consistent.

eFigure 1. Personal Impacts of Opioid Use Among Respondents with No Prescription or Illicit Opioid Use within Past 12 Months (N=1107)

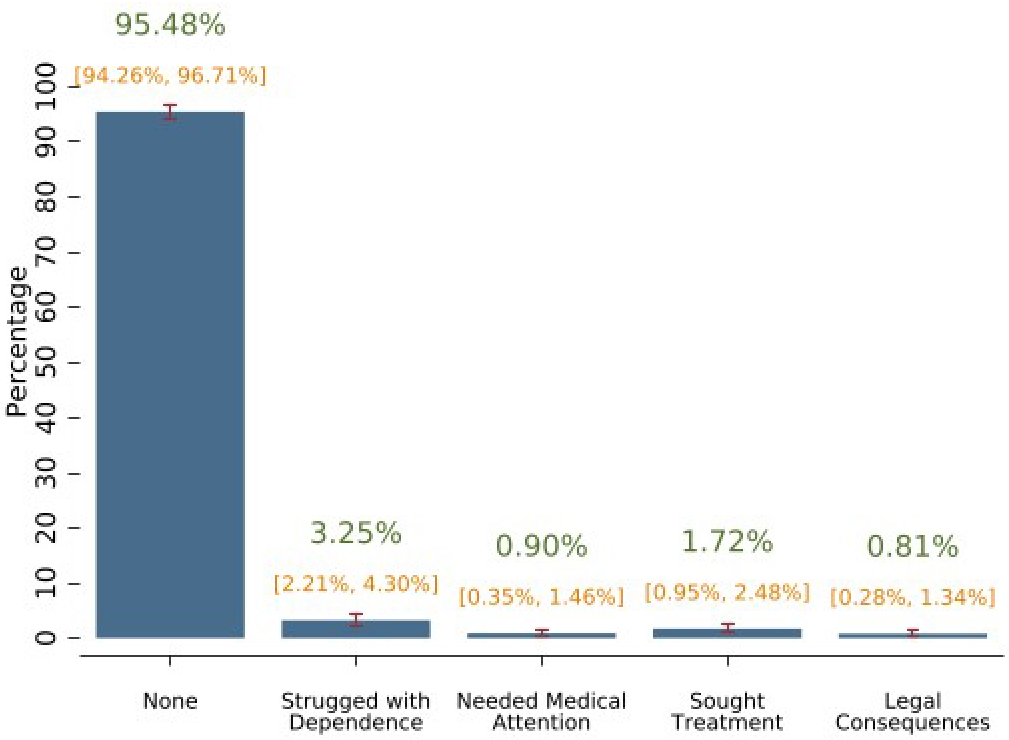

Notes: Respondents could select "None" or at least 1 of the other options.

eFigure 2. Self-Reported Likelihood of Overdosing from Opioid Use for Respondents with No Prescription or Illicit Opioid Use within Past 12 Months and No Personal Impact from Opioid Use (N=1057)

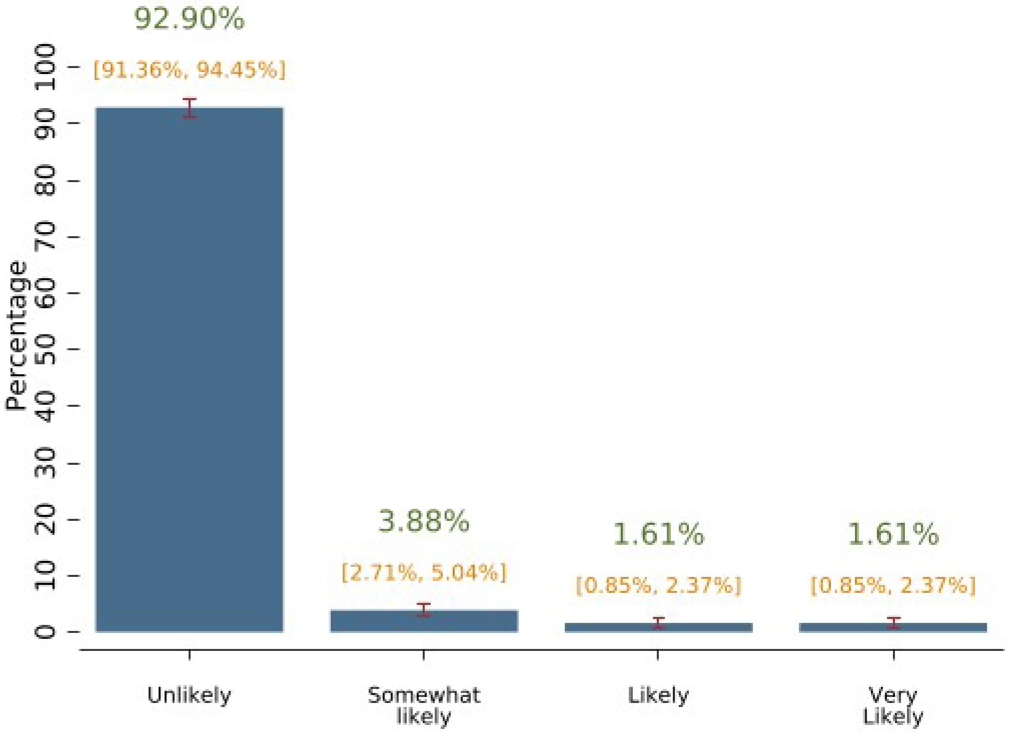

eTable. Demographic and Geographic Characteristics of the Survey Sample with Rates from the May 2024 Current Population Study

		Survey Sample	May 2024 Current Population Survey
**Gender**	Male	48.78%	48.74%
	Female	50.83%	51.26%
	Self-Identify	0.40%	
**Race**	Black	14.19%	12.96%
	White	71.75%	76.54%
	Other Race	14.06%	10.50%
**Ethnicity**	Hispanic	16.90%	17.95%
**Age**	18-24	12.28%	11.53%
	25-34	15.97%	17.15%
	35-44	21.58%	17.02%
	45-54	18.48%	15.45%
	55-64	18.55%	15.92%
	65-74	9.17%	13.48%
	75+	3.96%	9.45%
**Education**	No College	23.76%	38.15%
	Some College	36.17%	15.48%
	College Degree	40.07%	36.09%
**Region**	Midwest	21.65%	20.43%
	Northeast	18.22%	17.33%
	South	36.96%	38.64%
	West	23.17%	23.59%
**Sample Size**		1,515	79,350

Notes: The last column provides population shares using the May 2024 Current Population Study (CPS) and their population-weights. Educational attainment is summarized for the population ages 25+. Due to small sample sizes in our survey, we aggregated self-reported race into 3 categories.
