# Supplementary material for: Estimates of Illicit Opioid Use in the US
Source: JAMA Health Forum. 2025 May 9;6(5):e250809. doi: 10.1001/jamahealthforum.2025.0809 (PMC12065033; doi:10.1001/jamahealthforum.2025.0809)
Supplement: Supplement 2. — Data Sharing Statement [file jamahealthforum-e250809-s002.pdf]

Data Sharing Statement

Powell. Estimates of Illicit Opioid Use in the US. *JAMA Health Forum*. Published May 09, 2025. doi:10.1001/jamahealthforum.2025.0809

Data
Data available: Yes
Data types: Deidentified participant data
How to access data: We will submit all code and data to the Harvard Dataverse for public use.
When available: With publication

Supporting Documents
Document types: Statistical/analytic code
How to access documents: We will submit all code and data to the Harvard Dataverse for public use.
When available: beginning date: 07-01-2025

Additional Information
Who can access the data: All code and data will be public.
Types of analyses: All code and data will be made public for any use.
Mechanisms of data availability: We will submit all code and data to the Harvard Dataverse for public use.
Any additional restrictions: N/A
